# Infection with hypervirulent *Mycobacterium tuberculosis* triggers emergency myelopoiesis but not trained immunity

**DOI:** 10.3389/fimmu.2023.1211404

**Published:** 2023-06-13

**Authors:** Ana Raquel Maceiras, Diogo Silvério, Rute Gonçalves, Marcos S. Cardoso, Margarida Saraiva

**Affiliations:** ^1^ Instituto de Investigação e Inovação em Saúde (i3S), Universidade do Porto, Porto, Portugal; ^2^ Instituto de Biologia Molecular e Celular (IBMC), Universidade do Porto, Porto, Portugal; ^3^ Doctoral Program in Molecular and Cell Biology, Instituto de Ciências Biomédicas Abel Salazar (ICBAS), Universidade do Porto, Porto, Portugal

**Keywords:** tuberculosis, myelopoiesis, trained immunity, interferon-gamma, bone marrow

## Abstract

**Introduction:**

During infection, bone marrow (BM) hematopoiesis is reprogrammed toward myeloid cell production, a mechanism named emergency myelopoiesis. In addition to replenishing myeloid cells, emergency myelopoiesis has been linked to trained immunity, a process that allows enhanced innate immune responses to secondary challenges. Although hematopoietic alterations during tuberculosis (TB) have been described and *Mycobacterium tuberculosis* may colonize the BM, studies using the mouse model of infection and the laboratory reference strain *M. tuberculosis* H37Rv have demonstrated limited emergency myelopoiesis and trained immunity.

**Methods:**

To further address this issue, we aerosol- infected C57BL/6 mice with high doses of the hypervirulent M. tuberculosis isolate HN878 and monitored alterations to the BM. This experimental model better resembles the human blood immune signature of TB.

**Results and discussion:**

We found increased frequencies of lineage^-^Sca-1^+^cKit^+^ (LSK) cells and the granulocyte/macrophage progenitor (GMP) population. At the mature cell level, we observed an increase of monocytes and neutrophils in the blood and lung, likely reflecting the increased BM myeloid output. Monocytes or monocyte-derived macrophages recovered from the BM of *M. tuberculosis* HN878-infected mice did not show signs of trained immunity, suggesting an uncoupling of emergency myelopoiesis and trained immunity in the BM. Surprisingly, *M. tuberculosis* HN878-induced emergency myelopoiesis was not fully dependent on IFNγ, as mice lacking this cytokine and infected under the same conditions as wild-type mice still presented BM alterations. These data expand our understanding of the immune response to *M. tuberculosis* and raise awareness of pathogen strain-imposed differences to host responses.

## 1 Introduction

The bone marrow (BM) is highly responsive to stress cues, allowing the rapid adaptation of the hematopoietic process to ensure immune cell replenishment. This adaptation is key during infection and inflammation, in which a deviation of hematopoiesis toward the production of myeloid cells, a process also known as emergency myelopoiesis (EM), is well documented (1, 2). Recent evidence supports that, in addition to enhanced myeloid cell differentiation, EM plays a role in trained immunity (3), a mechanism that allows sustained innate immune responses to secondary challenges (4).

Hematopoietic adaptation has been reported in many infections, including those caused by Mycobacteria. Previous studies showed that, in the murine model, *Mycobacterium avium* infections enhanced the proliferation of hematopoietic stem cells (HSCs) (5) and that chronic *M. avium* infection caused pancytopenia (6) and anemia (7, 8). Furthermore, intradermal administration of *Mycobacterium bovis* BCG, the vaccine used against tuberculosis (TB), induced a transcriptomic rewiring of human stem and progenitor cells toward the myeloid cell lineage (9). In mice, intravenous administration of BCG caused emergency hematopoiesis and induced trained immunity in macrophages, which became highly protective against *Mycobacterium tuberculosis* (10). In the case of both *M. avium* and *M. bovis* BCG, interferon (IFN) γ was shown to be a key molecule in driving hematopoietic alterations (5, 6, 10, 11). The impact of IFNγ in myelopoiesis is further acknowledged in different scenarios (12–16). However, IFNγ has also been described as a negative modulator of HSC self-renewal, involved in BM failure (17), indicating that its role as a modulator of hematopoiesis is context dependent.

*M. tuberculosis* infections caused over 10 million new TB cases and killed over 1.6 million individuals in 2021 alone (18). Several observations support a possible impact of *M. tuberculosis* in the BM and in deregulating hematopoiesis. *M. tuberculosis* may persist in BM mesenchymal stem cells of TB patients even after antibiotherapy (19) and in long-term HSCs in individuals with latent TB infection (20). Additionally, clinical signs of abnormal hematopoiesis, such as anemia and thrombocytopenia, have been reported in TB patients (21, 22). Finally, neutrophilia and altered monocyte/T cell ratios are common in TB and are particularly associated with severe forms of the disease (23). Aerosol infection of mice with *M. tuberculosis* results in BM colonization (19, 24) and the increased recruitment of myeloid cells to the lungs (25), as well as increased numbers of blood monocytes, which has been shown to be caused by increased egress from the BM (26). Altogether, observations from human and mouse studies suggest the occurrence of EM during *M. tuberculosis* infections. However, a recent study showed that although *M. tuberculosis* reprogrammed HSCs in mice infected via aerosol, it also limited EM (24).

Here, we characterize the impact of *M. tuberculosis* infection on the BM using a mouse model previously shown to resemble the human blood immune signature (27). Specifically, we infected C57BL/6 mice with high doses of *M. tuberculosis* HN878 strain and analyzed the immune cell composition of the lung, blood, and BM at the peak of the disease. Our data show BM alterations compatible with the deviation of hematopoiesis toward myelopoiesis, with an increased output of myeloid cells in the blood and lungs of infected animals. Furthermore, we found that IFNγ may be dispensable to this process and despite the BM adaptation to infection, the generated monocytes or macrophages do not acquire an improved capacity to respond to secondary challenges.

## 2 Materials and methods

### 2.1 Animal housing, infection, and monitoring

C57BL/6 wild-type (WT) or C57BL/6.IFNγ deficient (IFNγ^-/-^) mice were bred and housed at the i3S Animal House Facility, and infected under ABSL3 conditions. Both male and female mice were used, ranging between 8 and 12 weeks of age at the time of infection. Animals were kept under a controlled temperature (20–24°C), humidity (45–65%), and light cycle (12h light/dark) in specific-pathogen free conditions. Water and food were provided *ad libitum*. Aerosol infection was performed using an inhalation exposure system (Glas-Col) (28, 29). Infection level was determined based on the lung bacterial burden 3 days post-infection: <200 CFUs corresponded to a low dose, while >500 CFUs was considered a high dose. Infected mice were weighed every week or every 2 days upon showing signs of disease. Weight loss of over 20% or lack of responsiveness to physical stimulation were considered humane endpoints, at which point the experiments were terminated. Mice were euthanized by CO_2_ inhalation, during which efforts were made to minimize suffering.

### 2.2 Organ processing

Organs were aseptically excised and processed as described previously (28, 29). Lungs were digested using Collagenase type IV (Gibco, Cat. #17104019) followed by physical disruption and filtering in a 70 µm mesh. Blood was collected via cardiac puncture. BM was collected by flushing the tibias and femurs with PBS + 2% FBS (Gibco, Cat. #10500064). Single-cell suspensions were used for bacterial burden determination, flow cytometry, and RNA analysis.

### 2.3 Bacteria growth and quantification

*M. tuberculosis* HN878 isolate was grown and stored as described previously (28, 29). Viable bacteria were determined by serial dilution and colony forming unit (CFU) enumeration after 21–28 days of incubation at 37°C in 7H11 agar plates (Cat. #212203, BD Biosciences). Bacterial quantification in infected lungs and BM was performed upon organ homogenization using the same procedure.

### 2.4 Flow cytometry

Mouse blood, BM, and lung cell suspensions were stained for surface antigens (30 min; 4°C) and fixed for 20 min in 4% paraformaldehyde-PBS after erythrocyte lysis. For the analysis of BM precursors, the mature lineage (Lin^+^) was depleted using an EasySep™ Mouse Streptavidin RapidSpheres™ Isolation Kit (Cat. #19860, STEMCELL Technologies) prior to Lin^-^ staining with specific antibodies for precursor and progenitor markers. Dead cells were excluded from the analyses using ZombieAqua (Cat. #423102, Biolegend) or ZombieGreen (Cat. #423112, Biolegend) viability dyes. Cells were acquired on a BD FACS Canto II or BD Fortessa II. Data were analyzed using *FlowJo* (version 10.1.r7). Dimensionality reduction of concatenated data was performed with the *Uniform manifold approximation and projection* (UMAP) *FlowJo* plug-in (version 3.1) (30). All antibodies used are listed in **Supplementary Table 1**. Gating strategies are shown in **Supplementary Figures 1A, B** and **Supplementary Figure 2A**.

### 2.5 RNA extraction and qPCR

Total RNA was extracted from mouse lung or BM cell suspensions using TripleXtractor (Cat. #GB23.0100, GRiSP) and converted to cDNA using a ProtoScript^®^ II First Strand cDNA Synthesis Kit (Cat. #E6560L, NE Biolabs). Relative *CSF2* gene expression quantification was performed using quantitative PCR and Taqman probe assay (Cat. #4331182; Assay ID #Mm01290062_m1, Thermo Fisher Scientific). Normalization of target gene abundance was performed using *Hprt* as the reference gene (Cat. #4331182; Assay ID #Mm03024075_m1, Thermo Fisher Scientific). Gene expression quantification for *Csf1*, *Csf3*, *Tnf*, *Il1b*, *Il6*, *Ifng*, and *Ifnb* was performed using quantitative PCR with SYBR green (Cat. #1725120, Bio-Rad) and intron spanning oligonucleotides specific for the target genes. Normalization of target gene abundance was performed using *Ubiquitin* as the reference gene. Melting and standard curves and RQ values were determined for each gene. Sequences of the oligonucleotides used are in **Supplementary Table 2**.

### 2.6 Histological analysis

Mouse lungs were fixed in 10% buffered formalin and embedded in paraffin. Serial 3-µm sections were used for Hematoxylin and Eosin (H&E) or immunofluorescence staining and analysis. Morphometric analysis of lung pathology areas in H&E-stained histological sections was performed using the software *Interactive Learning and Segmentation Toolkit* (*Ilastik* version 1.3.3) and *CellProfiler Analyst* (version 3.1.5). Probability maps for the whole lung and lesion area were created in Ilastik and calculated and analyzed using *CellProfiler Analyst* software (28). Pathological scoring analysis of H&E-stained histological sections was performed in a blind way, and the histopathological features were scored according to the parameters in **Supplementary Table 3**.

### 2.7 Immunofluorescence

Paraffin-embedded lung sections were stained for myeloperoxidase (Mpo) and nitric oxide synthase (Nos2), as described previously (28, 29, 31). Antigen recovery was performed by incubating slides at 96°C in HIER Buffer L (Cat. #TA135HBL, Epredia) for 45 min. Slides were then incubated overnight with goat anti-mouse Mpo (Cat. #AF3667, R&D systems; 1:40) and rabbit anti-mouse Nos2 (M-19, Cat. #sc-650, Santa Cruz Biotechnology, 1:50) at 4°C followed by incubation with secondary antibodies donkey anti-goat IgG Alexa Fluor 488 conjugated (Cat. #A-11055, Invitrogen) and donkey anti-rabbit IgG Alexa Fluor 647 conjugated (Cat. #A-31573, Invitrogen) for 2 h at room temperature. Slides were stained with DAPI (Cat. #422801, Biolegend; 1:1000 dilution), and glass coverslips were mounted using Fluoroshield (Cat. #F6182, Sigma-Aldrich). Images were acquired using IN Cell Analyzer 2000 (GE Healthcare) and analyzed using *IN Cell Developer software* (version 1.9) and *ImageJ* (version 1.53t) (32, 33).

### 2.8 Generation of bone marrow-derived macrophages and purification of bone marrow monocytes

BM-derived macrophages (BMDMs) were generated from BM cellular suspensions as previously described (29, 34). Cells were plated in Petri dishes and differentiated into macrophages using DMEM (Cat. #10566016, Gibco), completed with 1% sodium pyruvate (Cat. #S8636, Sigma-Aldrich), 1% HEPES (Cat. #15630106, Gibco), and 10% FBS (Cat. #10500064, Gibco) and supplemented with 20% L-cell conditioned medium (V/V). Once differentiation was complete (7 days), BMDMs were transferred to cell culture plates. BM monocytes were purified from BM cellular suspensions using an EasySep™ Mouse Monocyte Isolation Kit (Cat. #19861, STEMCELL Technologies), following the manufacturer’s protocol, and plated in cell culture plates with RPMI medium (Cat. #61870036, Gibco), completed as described above for DMEM. BMDMs were plated in 96-well plates (100,000 cells per well) for CFU determination or in 24-well plates (500,000 cells per well) for supernatant collection. Purified monocytes (purity >95%) were plated in 96-well plates (100,000 cells per well).

### 2.9 Human CD14^+^ cell isolation

Peripheral blood was collected and processed to obtain PBMCs as previously described (34). CD14^+^ monocytes were magnetically isolated using CD14 MicroBeads (Cat. #130-050-201, Miltenyi Biotec), following the manufacturer’s protocol. Purified cells (100,000 cells per well; purity >95%) were plated in 96-well plates with complete RPMI medium.

### 2.10 *In vitro* infections

Human CD14^+^ cells and mouse BMDMs or BM monocytes were infected with *M. tuberculosis* HN878 isolate at a multiplicity of infection (MOI) of 2 (2 bacteria:1 cell). Extracellular bacteria were washed away with PBS after 4 h and bacterial growth was quantified by CFU enumeration 4 h (control) or 4 days after infection. The culture supernatants (BMDMs and BM monocytes) were collected 24 h (in the absence of bacterial washing) after infection for protein quantification.

### 2.11 Cytokine quantification

Cytokine production by BMDM or monocyte cultures infected with *M. tuberculosis* was quantified by immunoassay, using commercially available ELISA kits (Cat. #88-7064-88; 88-7013-88; 88-7324-88, Invitrogen). 

### 2.12 Statistical analysis

Data were plotted and analyzed using *GraphPad Prism* (version 8.1.0): a non-parametric Mann–Whitney test was used to compare two groups and Kruskal–Wallis one-way analysis of variance test was used for the comparison of more than two groups. Multiple comparison correction was performed using a Dunn post-test. Significant differences are as follows: * p ≤ 0.05; ** p ≤ 0.01; and *** p ≤ 0.001. Spearman correlations and correspondent correlation plots were obtained using *R* (version 4.1.2) and *R* package *corrplot* (version 0.92).

## 3 Results

### 3.1 Infection with *M. tuberculosis* HN878 strain results in altered cell composition in the lung, blood, and bone marrow

**Figure 1:**
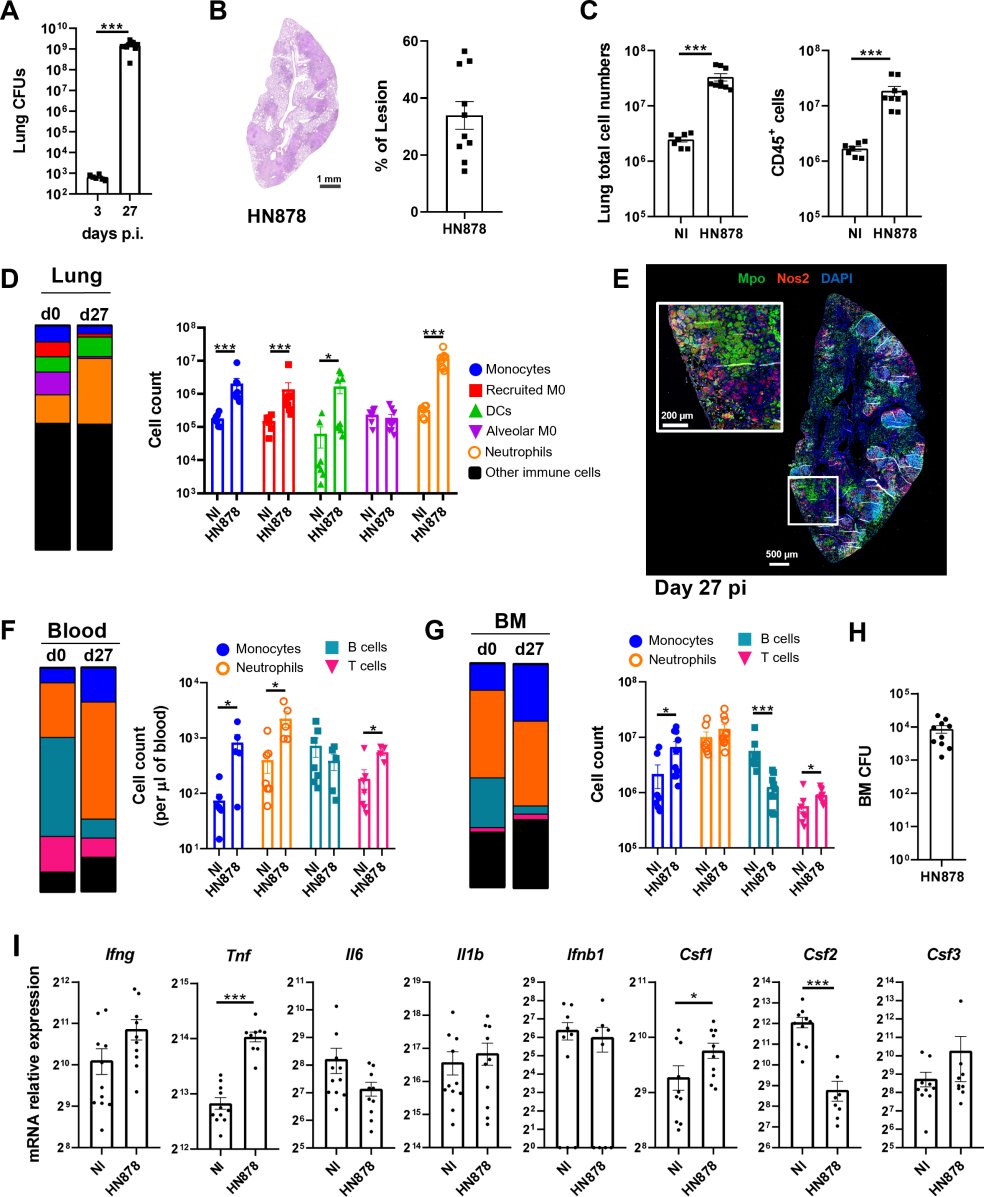
Infection with *M. tuberculosis* HN878 is associated with an accumulation of myeloid cells in the lung, blood, and bone marrow. C57BL/6 mice were aerosol-infected with high doses of *M. tuberculosis* isolate HN878. **(A)** Bacterial burden in the lungs determined on day 3 or 27 days post-infection. **(B)** Representative histological images of H&E-stained lung sections from non-infected (NI) or *M. tuberculosis*-infected (HN878) mice. Darker areas corresponding to tissue lesions were quantified to obtain the percentage of area of lesion in the lungs of *M. tuberculosis*-infected mice. **(C)** Number of total cells (left) and immune cells (right) in the lungs of NI or *M. tuberculosis*-infected mice. Immune cells were determined by FACS based on the detection of surface CD45. **(D)** Frequency (left) and total cell counts (right) of distinct myeloid cell populations: monocytes, recruited macrophages (recruited M0), dendritic cells (DCs), alveolar macrophages (alveolar M0), and neutrophils in NI or *M. tuberculosis*-infected mice. Gating strategies are shown in **Supplementary Figures 1A, B**. **(E)** Representative immunofluorescence image of a lung section from an infected mouse (27 days post-infection) stained for myeloperoxidase (Mpo) and nitric oxide synthase 2 (Nos2). Nuclei are stained with DAPI. **(F, G)** Frequency (left) and cell counts (right) of blood **(F)** or bone marrow **(G)** myeloid cell populations in NI or *M. tuberculosis*-infected mice. **(H)** Bacterial burdens in the bone marrow of mice 27 days post-infection. **(I)** Transcription analysis of the indicated genes detected by qPCR in the bone marrow of NI or *M. tuberculosis*-infected mice. Data are mean ± SEM. Each dot represents an individual mouse, with two independent experiments represented per panel. The infection doses (in CFU ± SEM)/animal ages (in weeks old at the time of infection) for the experiments used in this figure were 683 ± 125/10, 1,093 ± 100/8, and 1057 ± 74/9 and 10. Non-infected animals were 8 weeks old. The statistical analyses were performed using a Mann–Whitney U test to identify statistical differences between groups. p-values inferior to 0.05 were deemed significant: *p-value<0.05, ***p-value<0.001.

To investigate possible alterations imposed by *M. tuberculosis* infection to the BM hematopoietic output, we aerosol-infected WT C57BL/6 mice with high doses of *M. tuberculosis* HN878. Twenty-seven days post-infection (p.i.), the mice presented a high bacterial burden in the lungs (over 10^9^ bacteria) (**Figure 1A**), which was accompanied by marked tissue pathology, with lesions affecting 14% to 56% of the lung (**Figure 1B**). Additionally, we observed an increase in lung cellularity, associated with the recruitment and accumulation of CD45^+^ immune cells (**Figure 1C**). We next analyzed by flow cytometry the myeloid cell composition within the immune cells present in the lung, which accounted for approximately 45% of the total CD45^+^ cells (**Figure 1D**; **Supplementary Figure 1A**). Although the frequency of alveolar macrophages decreased with infection, resulting in a very low percentage 27 days p.i., both the frequency and numbers of all other myeloid cell types (monocytes, monocyte-derived recruited macrophages, dendritic cells (DCs), and neutrophils) were significantly increased in infected mice (**Figure 1D**). Neutrophils exhibited the highest increase both in percentage and cell counts of the myeloid populations analyzed. The global frequency of other immune cells (which included eosinophils and NK, T, and B cells) was not altered (**Figure 1D**). Immunofluorescence staining of lung sections with Mpo, a neutrophil marker, revealed large Mpo-positive areas, which were dominant compared with those corresponding to Nos2-positive activated macrophages (**Figure 1E**). These results are in line with previous reports showing an accumulation of myeloid cells, especially neutrophils, in the lungs upon infection with *M. tuberculosis* HN878 (28, 31).

We then analyzed the blood and BM compartments at 27 days p.i. (**Supplementary Figure 1B**). Infected mice presented leukocytosis (**Supplementary Figure 1C**), which was associated with increased numbers (per µl of blood) of monocytes, neutrophils, and T cells, even though the proportion of T cells in the blood had diminished (**Figure 1F**). There was a reduction in the frequency of B cells, with no effect on cell numbers (**Figure 1F**). Thus, the increase in myeloid cells seen in the lungs of infected mice was also observed in the blood, suggesting a possible altered hematopoietic output. Analysis of the BM mature cell populations showed an increase in the frequency and numbers of monocytes, which was accompanied by a decrease in the frequencies and numbers of B cells (**Figure 1G**). Both neutrophils and T cells remained mostly unaltered (**Figure 1G**), as did the total number of BM cells (**Supplementary Figure 1D**). Importantly, *M. tuberculosis* disseminated into the BM (**Figure 1H**). Targeted transcriptional analysis of the BM 27 days p.i. showed a modest impact of infection on the expression of cytokine genes involved in hematopoietic reprogramming. *Tnf* and *Csf1* expression was statistically significantly increased and *Csf2* expression was statistically significantly decreased (**Figure 1I**). The lack of more profound differences in gene expression and the decrease of *Csf2* transcription upon infection was surprising and may reflect the overall exhaustion of the infected BM.

The accumulation of myeloid cells in the lungs of the infected animals, together with the alterations seen in the blood and BM, raise the hypothesis that EM may be happening in this immune compartment.

### 3.2 *M. tuberculosis* HN878 infection leads to bone marrow myelopoiesis

**Figure 2:**
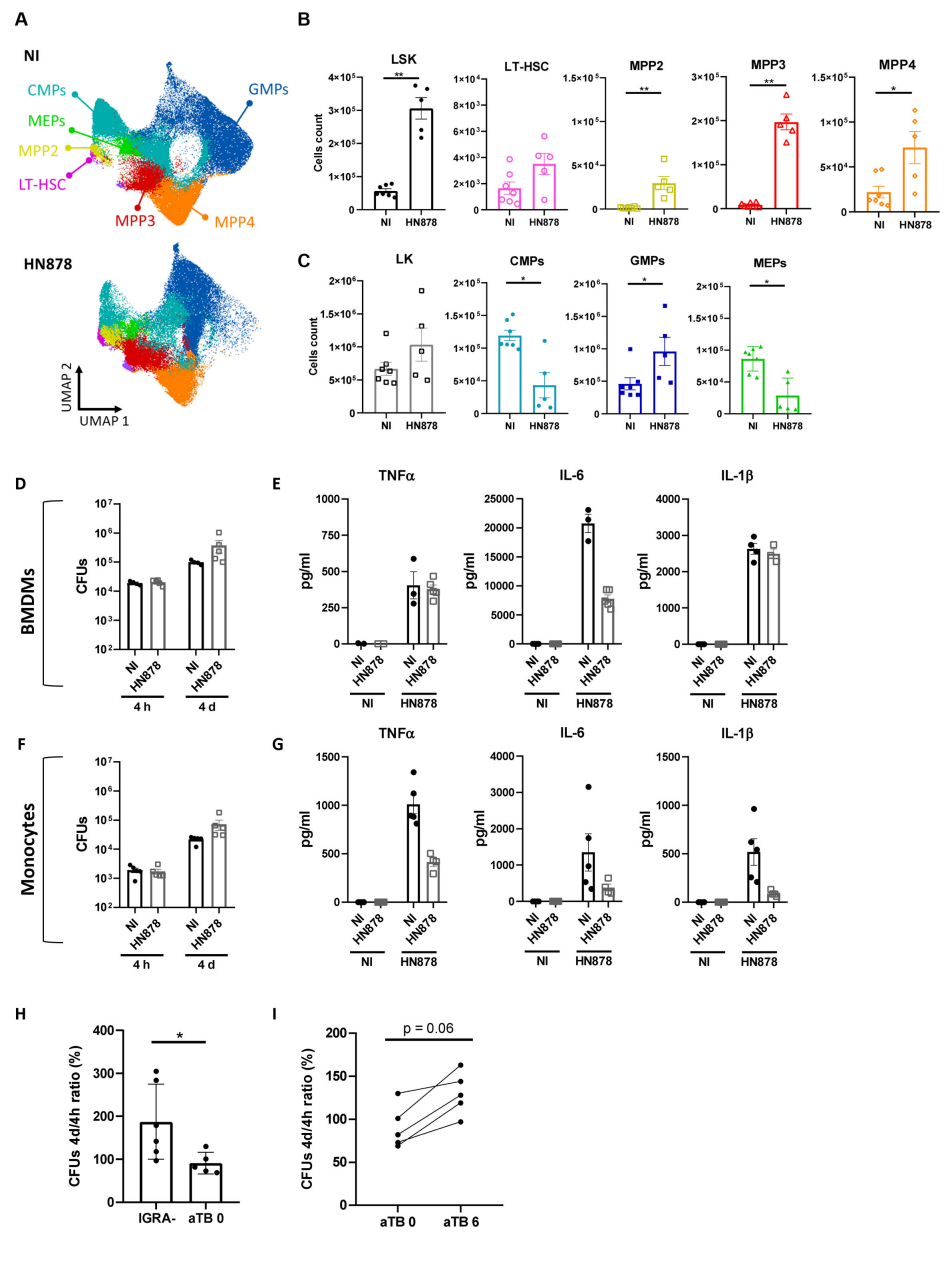
Aerosol infection with high doses of *M. tuberculosis* HN878 leads to emergency myelopoiesis but does not result in trained immunity. Bone marrow cells of C57BL/6 mice aerosol-infected with high doses of *M. tuberculosis* isolate HN878 were recovered and analyzed by flow cytometry **(A–C)** or used to investigate trained immunity **(D–G)**. Non-infected (NI) mice (8 weeks old) were used as controls. **(A)** UMAP of flow cytometry data overlaying different immune progenitor populations: CMPs (CD34^hi^FcγR^-^), GMPs (CD34^hi^FcγR^+^), MEPs (CD34^lo^FcγR^-^), LT-HSC (Flt3^-^CD150^+^CD48^-^), MPP2 (Flt3^-^CD150^+^CD48^+^), MPP3 (Flt3^-^CD150^-^CD48^+^), and MPP4 (Flt3^+^CD150^-/int^). **(B)** Total cell count of LSK (Lin^-^Sca1^+^c-Kit^+^) progenitor and LSK cell subpopulations: LT-HSC, MPP2, MPP3, and MPP4. **(C)** Total counts of LK (Lin^-^Sca1^-^c-Kit^+^) cells and LK subpopulations: CMPs, GMPs, and MEPs, in the bone marrow. Gating strategies are shown in **Supplementary Figure 2A**. Bone marrow-derived macrophages were generated from NI (closed circles) or *M. tuberculosis* HN878-infected (open squares) mice. Mature cells (day 7 of culture) were infected with the same isolate of *M. tuberculosis* at an MOI of 2. **(D)** Bacterial burdens were determined by CFU enumeration after 4 h and 4 days of infection. **(E)** The production of cytokines TNFα, IL-6, and IL-1β was measured in non-stimulated (NS) or infected (HN878) cell cultures by immunoassay 24 h post-infection. Monocytes were isolated from the bone marrow of NI (closed circles) or *M. tuberculosis*-infected (open squares) mice for **(F)** CFU determination **(F)** or protein analysis **(G)**, as before. **(H)** Monocytes (CD14^+^) were isolated from the blood of IGRA^−^ healthy donors or active TB patients at presentation (aTB 0) or after 6 months of antibiotherapy (aTB 6), and infected with *M. tuberculosis* for 4 h or 4 days, for bacterial burden determination. The percentage increase of CFU from 4h to 4 days of *in vitro* infection is shown. Data are mean ± SEM. Each dot represents an individual mouse, distributed in two independent experiments represented per **(B–G)** or an individual donor **(H, I)**. The infection doses (in CFU ± SEM)/animal ages (in weeks old at the time of infection) for the experiments used in this figure were 1,093 ± 100/8 and 650 ± 110/8. The statistical analyses were performed using a Mann–Whitney U test to identify statistical differences between groups. p-values inferior to 0.05 were deemed significant: *p-value<0.05 and **p-value<0.01.

To investigate whether EM may be occurring during *M. tuberculosis* HN878 infections, we analyzed the BM progenitor populations of naive versus HN878-infected mice 27 days p.i. (**Supplementary Figure 2A**). UMAP dimensionality reduction analysis of flow cytometry data revealed a different precursor and progenitor cell composition profile between infected and non-infected mice (**Figure 2A**). Closer analysis of the different precursor populations revealed a statistically significant increase of LSK (Lin^-^Sca^+^c-Kit^+^) cells in infected mice (**Figure 2B**). Within the LSK population, long-term HSC (LT-HSC) were not affected by the infection, but an increase of the multipotent progenitors (MPP) 2, 3, and 4 was observed (**Figure 2B**). Moreover, although no statistically significant differences were observed for the frequencies of LK (Lin^-^Sca^-^c-Kit^+^) cells (**Figure 2C**), there was a statistically significant increase in granulocyte-monocyte progenitors (GMPs) accompanied by a decrease in common myeloid progenitors (CMPs) and megakaryocyte erythrocyte progenitors (MEPs) (**Figure 2C**). Similar alterations were seen in the frequencies of precursor and progenitor populations (**Supplementary Figures 2B, C**). Altogether, these data suggest the occurrence of EM as a consequence of aerosol infection with high doses of *M. tuberculosis* HN878.

Given the apparent discrepancy between these data and previously published data (24), we decided to infect mice with low doses of *M. tuberculosis* HN878. Under these conditions and at day 34 p.i., we still observed colonization of the BM (**Supplementary Figure 2D**), although the bacterial burden was 2 log lower than that resulting from infections with high doses of bacteria. Aerosol infection with low doses of *M. tuberculosis* HN878 did not significantly remodel BM cell composition, as no changes in mature cell populations were observed (**Supplementary Figure 2E**), and only an increase in LSK (**Supplementary Figure 2F**) and a modest decrease in CMPs (**Supplementary Figure 2G**) were observed. Interestingly, a positive correlation was observed between the BM LSK population and lung bacterial burden, monocytes, and neutrophils, as well as BM bacterial burden (**Supplementary Figure 2H**).

Collectively, our findings raise the hypothesis that EM may occur during *M. tuberculosis* infections, possibly associated with more severe outcomes of disease, such as those resulting from high doses of infection.

### 3.3 *M. tuberculosis* impact on the bone marrow does not translate into trained immunity

As we observed the adaptation of the BM towards myelopoiesis in our model of aerosol infection with high doses of *M. tuberculosis* HN878, we next investigated whether the resulting mature cells might be trained. As such, we generated macrophages derived from the BM of non-infected or *M. tuberculosis*-infected mice for 27 days. The resulting BMDMs were infected with *M. tuberculosis* HN878 and the bacterial burden was measured 4 h and 4 days p.i. The bacterial burden 4 h after infection was similarly independent of the BMDM origin. Then, an increase in intracellular bacteria was observed over time and was similarly independent of the BMDM origin (**Figure 2D**), suggesting that previous infection with *M. tuberculosis* did not enhance the protective capacity of *de novo*-generated BMDM. Additionally, we measured the production of TNFα, IL-6, and IL-1β, cytokines associated with trained immunity in the context of mycobacteria (35, 36), by BMDMs generated from non-infected or infected mice and *in vitro* infected with *M. tuberculosis* HN878. The ability of the generated BMDMs to respond to secondary stimuli was also not enhanced by previous infection across the measured parameters (**Figure 2E**). Finally, to exclude possible effects imposed on macrophages by the *in vitro* differentiation process, we purified monocytes from the BM of non-infected or *M. tuberculosis*-infected mice and analyzed their microbicidal capacity (**Figure 2F**) and cytokine response to secondary stimuli (**Figure 2G**). Monocytes purified from non-infected or infected mice responded similarly, further supporting that despite evidence of EM in the BM, this was not accompanied by training of the mature populations.

As trained immunity was suggested to equip circulating monocytes from individuals recently exposed to *M. tuberculosis* with an increased capacity to control BCG (37), we considered the hypothesis that trained immunity may occur at the periphery. To test this hypothesis, we started by comparing the bacterial growth over a 4-day period in monocytes isolated from IGRA**^−^** or active TB patients. In IGRA**^−^** monocytes the bacteria were able to grow; however, this growth was much more limited in the case of active TB monocytes (**Figure 2H**). Moreover, when we compared the ability of monocytes from active TB patients to control *M. tuberculosis* before and after antibiotherapy, we found that after treatment the growth of bacteria was superior for all tested donors (**Figure 2I**).

Altogether, our data demonstrate that in a mouse model mimicking the human transcriptional signature of TB, BM monocytes or macrophages did not acquire trained immunity despite the occurrence of EM. However, untreated active TB in humans appeared to associate with circulating monocytes that display an increased capacity to control *M. tuberculosis* growth in *in vitro* infections.

### 3.4 Impact of IFNγ deficiency on emergency myelopoiesis upon infection with *M. tuberculosis* HN878

**Figure 3:**
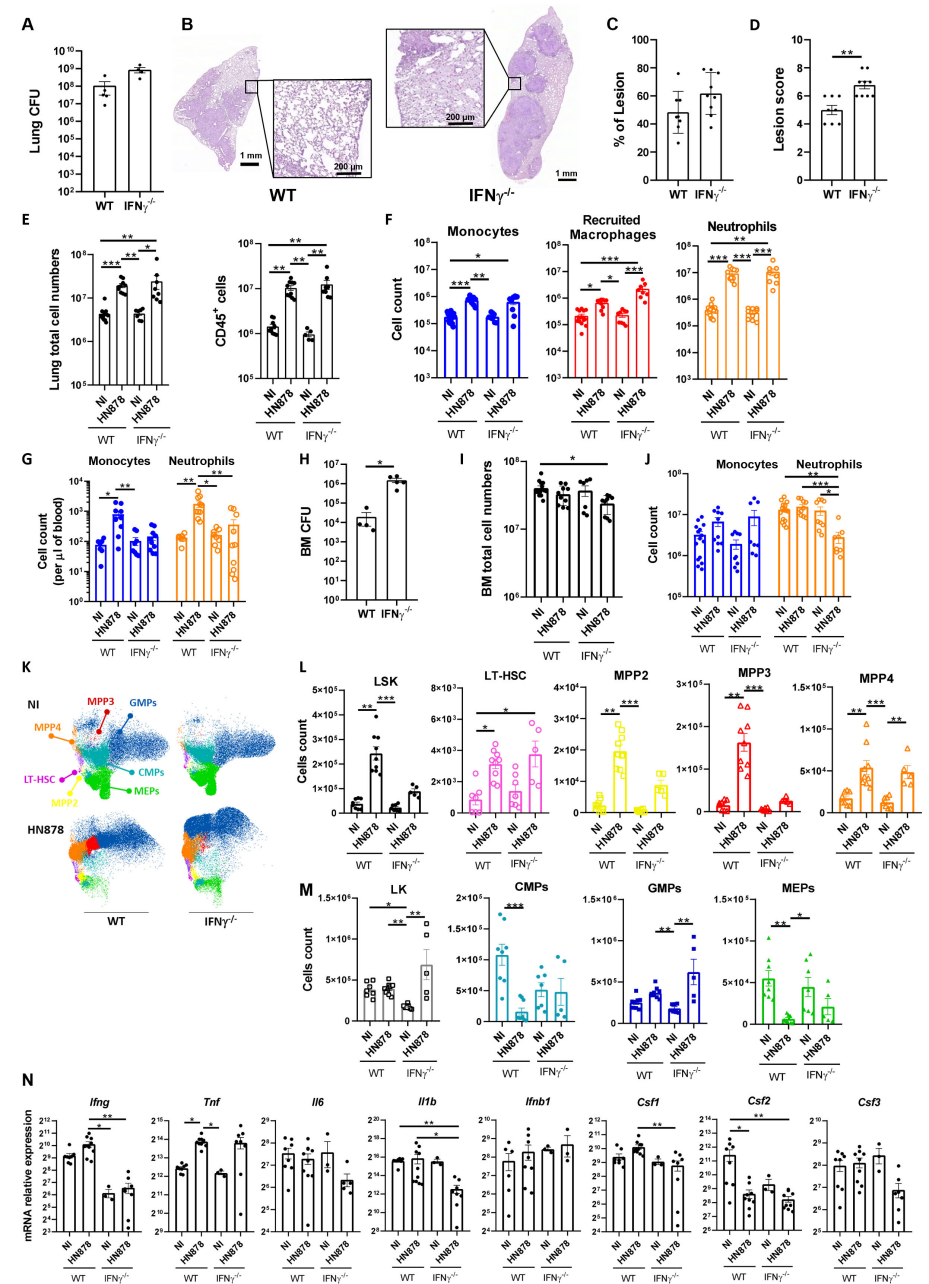
Role of IFNγ in *M. tuberculosis*-induced emergency hematopoiesis. C57BL/6 WT or IFNγ^-/-^ mice were aerosol-infected with high doses of *M. tuberculosis* isolate HN878. When animals reached humane endpoints the experiment was terminated. This happened between days 24 and 27 post-infection. At these time points, the bacterial burden in the lungs was determined **(A)**. **(B)** Representative histological images of H&E-stained lung sections from *M. tuberculosis*-infected WT or IFNγ^-/-^ mice. Zoomed areas show lesion-adjacent tissue. **(C)** Morphometric quantification of the percentage of lesion in the lung section. **(D)** Histopathological score attributed to each lesion. **(E)** Number of total cells (left) and immune cells (right) in the lungs of NI or *M. tuberculosis*-infected mice. Immune cells were determined by FACS based on the detection of surface CD45. **(F)** Number of monocytes, recruited macrophages, and neutrophils in NI or *M. tuberculosis*-infected mice. Gating strategies are shown in **Supplementary Figures 1A, B**. **(G)** Monocyte and neutrophil cell counts per µl of blood of NI or *M. tuberculosis*-infected mice. **(H)** Bacterial burdens in the bone marrow of WT or IFNγ^-/-^ mice 24–27 days post-infection. **(I, J)** Total cell numbers **(I)** and numbers of monocytes and neutrophils **(J)** in the bone marrow of NI and infected mice. **(K)** UMAP of flow cytometry data overlaying different immune progenitor populations: CMPs (CD34^hi^FcγR^-^), GMPs (CD34^hi^FcγR^+^), MEPs (CD34^lo^FcγR^-^), LT-HSC (Flt3^-^CD150^+^CD48^-^), MPP2 (Flt3^-^CD150^+^CD48^+^), MPP3 (Flt3^-^CD150^-^CD48^+^), and MPP4 (Flt3^+^CD150^-/int^). **(L)** Total cell count of LSK (Lin^-^Sca1^+^c-Kit^+^) progenitor and LSK cell subpopulations: LT-HSC, MPP2, MPP3, and MPP4. **(M)** Total counts of LK (Lin^-^Sca1^-^c-Kit^+^) cells and LK subpopulations: CMPs, GMPs, and MEPs, in the bone marrow. Gating strategies are shown in **Supplementary Figure 2A**. **(N)** Transcription analysis of the indicated genes detected by qPCR in the bone marrow of NI or *M. tuberculosis*-infected WT or IFNγ^-/-^ mice. Data are mean ± SEM. Each dot represents an individual mouse, distributed in two independent experiments represented per panel. The infection doses (in CFU ± SEM)/animal ages (in weeks old at the time of infection) for the experiments used in this figure were 1,093 ± 100/8 for WT and 11–12 for IFNγ^-/-^ and 1,057 ± 74/9–10 for WT and 9 for IFNγ^-/-^. Non-infected animals were 10–11 weeks old. The statistical analyses were performed using a Mann–Whitney U test when comparing only two groups, or a Kruskal–Wallis one-way analysis of variance to identify statistical differences between groups. p-values inferior to 0.05 were deemed significant: *p-value<0.05, **p-value<0.01, and ***p-value<0.001.

As IFNγ has been widely implicated in EM (38, 39) and we observed an increase of the expression of this cytokine in the BM of *M. tuberculosis*HN878-infected mice (**Figure 1I**), we sought to investigate whether its presence was required for the EM observed in the C57BL/6 model of infection. Thus, we aerosol-infected C57BL/6 WT and IFNγ^-/-^ mice with a high dose of *M. tuberculosis* HN878. IFNγ^-/-^ mice presented higher lung bacterial burdens than WT mice, in agreement with their known inability to cope with *M. tuberculosis* infections (**Figure 3A**). In the same line, histological analysis revealed more extensive lesions in IFNγ^-/-^ mice than in WT mice, with damaged tissue and pulmonary exudate visible in areas adjacent to lesions (**Figure 3B**). Both the percentage of lesion (**Figure 3C**) and the lesion score (**Figure 3D**) were increased in infected IFNγ^-/-^ mice compared with WT mice. A lack of IFNγ did not impact the number of total or immune (CD45^+^) lung cells (**Figure 3E**). The numbers of monocytes, recruited macrophages, and neutrophils present in the lungs of IFNγ competent or deficient mice were also similar (**Figure 3F**). Despite this overall similarity in myeloid cell composition in the lungs of infected WT and IFNγ^-/-^ mice, the increase of monocytes and neutrophils seen in the blood of infected WT mice was not observed in the absence of IFNγ (**Figure 3G**). We then focused on analyzing the BM compartment. As observed in the lung, IFNγ^-/-^ mice presented a higher bacterial burden in the BM than WT mice (**Figure 3H**), with BM cellularity (**Figure 3I**) and the variations in the number of monocytes (**Figure 3J**) similar in the two mouse strains. However, IFNγ^-/-^ mice exhibited a reduction in BM neutrophils compared with WT mice (**Figure 3J**). These data indicate that, in the absence of IFNγ, the accumulation of monocytes and neutrophils observed in the lung is not fully reflected in the blood and the BM, as seen for WT mice. Although this finding is not fully understood, it may suggest a faster recruitment of neutrophils to the lung, with increased exit rates from the BM, or alternatively an increased lifespan of neutrophils in the lung.

Next, we questioned whether EM would occur in IFNγ^-/-^ mice upon *M. tuberculosis* HN878 infection. Similar to the WT controls, UMAP reduction analysis of flow cytometry data disclosed differences in precursor and progenitor cell composition profiles between infected and non-infected IFNγ^-/-^ mice (**Figure 3K**). The analysis of precursor populations revealed an overall similar tendency between WT and IFNγ^-/-^ mice upon infection, with an increase of all populations in infected mice compared with uninfected mice (**Figure 3L**). However, for some populations, such as LSK, MPP2, and MPP3, this increase was less marked in the case of IFNγ^-/-^ mice, (**Figure 3L**). A similar profile was observed in progenitor populations, with less pronounced alterations seen in IFNγ^-/-^ mice (**Figure 3M**). Of note, the most marked change observed in IFNγ^-/-^ mice upon infection was an increase in the GMP population (**Figure 3M**). Similar findings were observed in terms of cell population frequencies (**Supplementary Figure 3**). Altogether, these findings suggest that EM in response to severe *M. tuberculosis* infections might occur, at least to some extent, even in the absence of IFNγ. EM has been linked to inflammatory environments in the BM (40). Thus, we questioned whether the lack of IFNγ would alter the inflammatory environment seen in the BM of infected WT mice (**Figure 1I**). Interestingly, the transcription of several cytokine genes in the BM was decreased in infected IFNγ^-/-^ mice compared with WT or uninfected mice (**Figure 3N**). However, this was not the case for TNF (**Figure 3N**), the transcription of which was enhanced by the infection independently of IFNγ (**Figure 3N**).

Together these data suggest that IFNγ is partly dispensable for the occurrence of EM upon the infection of mice with *M. tuberculosis* HN878, consequently suggesting the occurrence of alternative mechanisms.

## 4 Discussion

TB remains a major health problem, with increasing numbers of new cases and fatalities reported over the past 2 years (18). Neutrophils have been consistently associated with poor outcomes of TB in humans and experimental models of TB (41, 42). Accompanying the enhanced neutrophil blood signature present in TB patients (43), an under-abundance of B cell, NK cell, and effector T cell signatures was also identified in active TB, progression from latent TB, and TB-susceptible mice (27). Decreased levels of mRNAs associated with lymphocyte lineages and increased expression of those associated with myeloid differentiation were also associated with progression from latent to active TB (44). Thus, a myeloid-biased remodeling of the hematopoietic landscape likely accompanies TB disease progression and establishment, which may at least partially result from the adaptation of the BM to the infection, as seen in other infection and inflammatory settings (2, 45). To experimentally address this hypothesis, we characterized the alterations imposed by *M. tuberculosis* infection on the mature and progenitor cellular populations in the BM. As such, C57BL/6 mice were aerosol-infected with high doses of *M. tuberculosis* isolate HN878. This experimental condition results in severe TB disease and is the one condition to better recapitulate the blood transcriptomic signatures of TB patients in the mouse C57BL/6 genetic background (27). The *M. tuberculosis* HN878 isolate has been described as hypervirulent as a result of enhanced induction of type I IFN and deregulated Th1 immunity (46, 47). More recently, infections with *M. tuberculosis* HN878 were also shown to lead to neutrophil overactivation by type I IFN, with exacerbated NETosis formation and extensive tissue pathology (31).

We observed alterations to the BM cell progenitor composition, namely an increased frequency of LSK and GMP cell populations, together with a decrease in the CMP population. Furthermore, we also observed an expansion of monocytes in the BM, blood, and lung and of neutrophils in the blood and lung. Altogether, our data support the occurrence of EM as a consequence of *M. tuberculosis* infection. Additionally, a previous study identified an increase in the LSK population but not in GMPs upon *M. tuberculosis* infection (24). However, in that study, a different strain of *M. tuberculosis*, the laboratory reference strain H37Rv, was used and the aerosol infection was performed with low bacilli doses, which results in a milder outcome of infection, with the mice surviving for long time periods and presenting lower bacterial burdens in the lungs and BM (24). Most notably, the blood transcriptional signature observed in TB patients was not reproduced in such experimental conditions (27). Therefore, it is likely that the discrepancies seen in terms of GMP dynamics upon infection are reflecting the severity of the infection and contributing to the characteristic TB blood immune signature. In line with this, we show that infections of mice with low doses of *M. tuberculosis* HN878 did result in BM colonization but not in EM. Additionally, it is important to emphasize that infections with the *M. tuberculosis* HN878 isolate, such as those performed in this study, induce a relatively rapid progression to disease and may thus be reflecting the peak of active TB, rather than a long-term chronic infection. The differences seen in the GMP dynamics between high and low doses of infection, or between isolates of distinct virulence, may also reflect BM adaptation to these two different settings: peak of TB disease vs. chronic TB infection.

Independently of the distinct BM adaptation to infections by the different *M. tuberculosis* isolates, a common point is the resulting impaired trained immunity. In fact, we observed decreased cytokine production in response to secondary challenge by monocytes isolated from infected BM. It is possible that the environment the BM is subjected to by hypervirulent strains of *M. tuberculosis* may promote innate cell tolerance. This is an intriguing question worth investigating in the future. Interestingly, trained immunity has previously been suggested to equip monocytes from individuals recently exposed to *M. tuberculosis* with increased capacity to control BCG (37). By comparing the ability of monocytes purified from IGRA**^−^** donors or from active TB patients at presentation or post-treatment, we also report that the best controllers of *M. tuberculosis* growth are monocytes from active TB patients before treatment. Therefore, it is tempting to speculate that training of myeloid cells in the context of *M. tuberculosis* may be achieved in the periphery rather than in the BM. Related to this, training of alveolar macrophages has been described in several studies, namely upon viral infection (48–50). Further exploring these questions using genetically distinct isolates of *M. tuberculosis* and strains of mice is exciting, as uncovering potential mechanisms of trained immunity in circulating monocytes and resident macrophages may unleash novel strategies to tackle TB.

The mechanisms underlying BM adaptation during TB, and how they may articulate with immunity at the site of infection, remain elusive. In line with several previous studies (19, 20, 24), we show colonization of the BM by *M. tuberculosis*. Thus, the presence of the bacteria in the BM may directly impact on the hematopoietic skewing, e.g., by activating pattern recognition receptors expressed on HSCs or progenitor cells (2, 45). On the other hand, the local and systemic inflammatory response caused by the infection may play a key role in modulating the hematopoietic niche and prioritizing myelopoiesis. Indeed, several cytokines have been described to modulate myelopoiesis, including type I and type II IFNs, IL-1, TNFα, IL-6, IL-27, and colony stimulating factors (2, 45). Analysis of the transcription of several of these molecules in the BM upon infection with high doses of *M. tuberculosis* HN878 revealed only modest changes. However, as we are analyzing bulk RNA and one time point only, we cannot exclude an elevation of some of these or other molecules in the BM niche over time. Of note, IFNγ has been shown to play such a role in the context of *M. avium* and *M. bovis* BCG infections (5, 10), but not in other settings (17), which suggests a context-dependent action. In the context of aerosol infection with high doses of *M. tuberculosis* HN878, IFNγ appears to be partly dispensable. It is possible that the excessive inflammation resulting from infections by *M. tuberculosis* HN878 may surpass on one hand the requirement of IFNγ for myelopoiesis, as seen in BCG (10), and on the other hand, the limiting effect of type I IFN, as seen in *M. tuberculosis* H37Rv infections (24). Whether the global alteration to the hematopoietic niche or a particular cytokine are key effectors in our setting remains to be addressed. The most marked alteration in the BM of infected IFNγ^-/-^ mice was the pronounced increase of GMPs. This is interesting because even though neutrophils accumulated in the lungs of these mice, an increase of these cells in the blood was not observed. In addition to the differential recruitment of neutrophils to the lungs of IFNγ^-/-^ mice, it is also conceivable that the full differentiation of GMPs is not achieved in the absence of IFNγ. Finally, it is worth mentioning that compared with WT mice, IFNγ^-/-^ mice show increased lung bacterial burdens and lesion scores in the absence of increased cellularity. It is possible that a lack of IFNγ in infections with *M. tuberculosis* HN878 may not impact the already high amount of neutrophils in the lung described for this isolate (31) but contribute to the worsening of lesions by failing to control neutrophil responses, as described in the context of *M. tuberculosis* H37Rv infections (51).

In conclusion, this study adds to the expanding evidence linking BM alterations to myeloid/lymphoid imbalances in TB. Understanding the crosstalk between central hematopoiesis and local immune responses may provide novel insights into the immune response to TB, which is an important driver of TB disease. This may in turn benefit the design of novel host-directed therapies (52, 53), which are acknowledged as promising strategies to mitigate TB severity and mortality.

## Data availability statement

The original contributions presented in the study are included in the article/**Supplementary Material**. Further inquiries can be directed to the corresponding author.

## Ethics statement

The study protocol leading to peripheral blood mononuclear cells (PBMC) isolation was approved by the Health Ethics Committees of the Centro Universitário Hospitalar de S. João (approval number 109-11), the North Health Region Administration (approval number 71-2014) and the Portuguese Data Protection Authority (approval number 12174-2011). To ensure confidentiality, each case was anonymized by the assignment of a random identification number. Participants were enrolled at a TB clinic in Porto and provided informed consent. Experiments were conducted according to the principles expressed in the Declaration of Helsinki. The patients/participants provided their written informed consent to participate in this study. Animal experiments followed the 2010/63/EU Directive and were approved by the i3S Animal Ethics Committee and the Portuguese National Authority for Animal Health (DGAV; #018413/2021-11-24).

## Author contributions

AM, DS, and MS designed the experiments, analyzed the data, and wrote the manuscript; AM, DS, RG, and MC performed the experiments; MS obtained funding. All authors contributed to the article and approved the submitted version.
