# Supplementary material for: Infection with hypervirulent Mycobacterium tuberculosis triggers emergency myelopoiesis but not trained immunity
Source: Front Immunol. 2023 Jun 13;14:1211404. doi: 10.3389/fimmu.2023.1211404 (PMC10296772; doi:10.3389/fimmu.2023.1211404)
Supplement: Supplementary file 1 [file DataSheet_1.docx]

***Supplementary Material***

Infection with hypervirulent *Mycobacterium tuberculosis* triggers emergency myelopoiesis, but not trained immunity

**Ana Raquel Maceiras, Diogo Silvério, Rute Gonçalves, Marcos Cardoso, Margarida Saraiva^*^**

*** Correspondence:**

Margarida Saraiva

margarida.saraiva@ibmc.up.pt

**
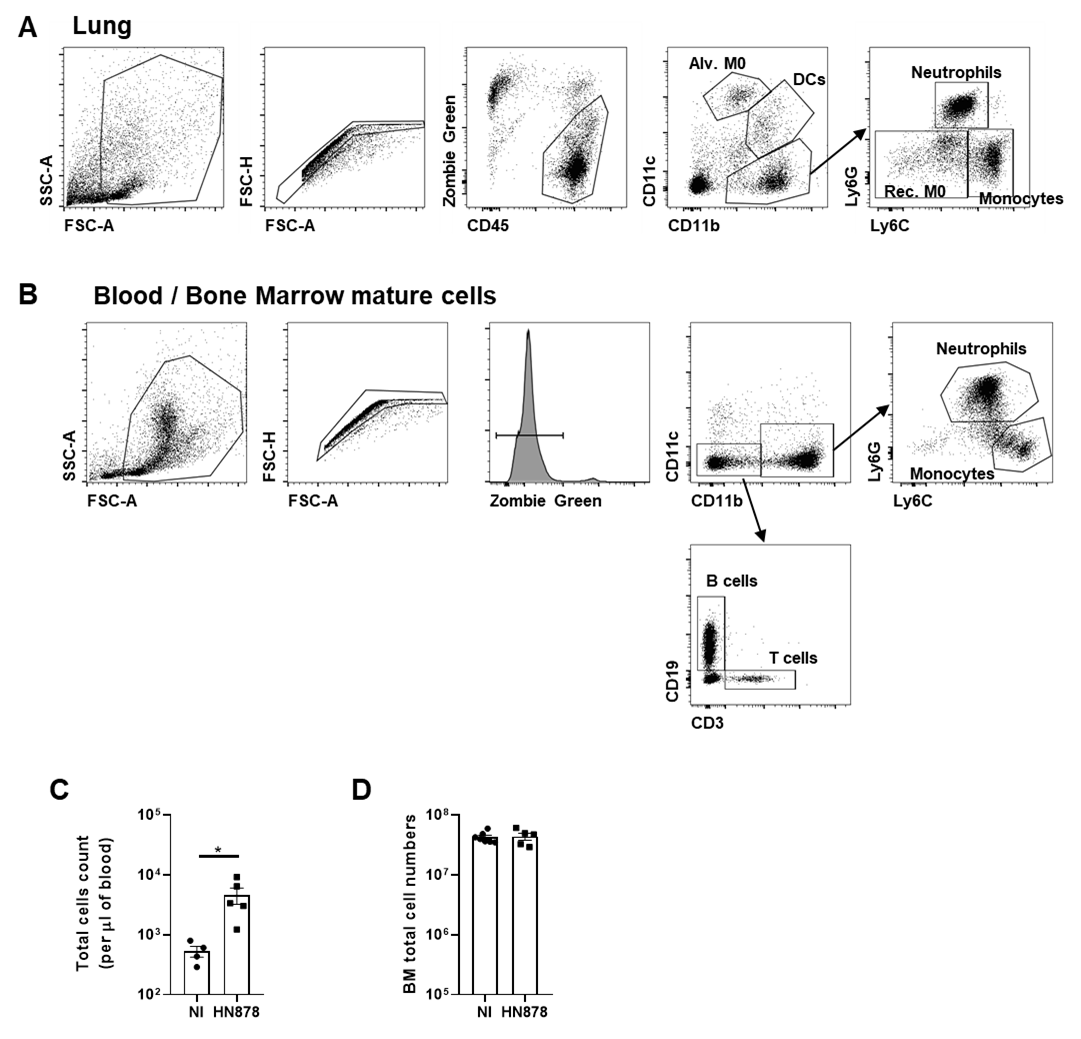
**

**Supplementary Figure 1.** Gating strategy used to analyse lung (A) and blood and bone marrow (B) immune mature cell populations. Total cell numbers present in the blood (C) or the bone marrow (D) of non-infected (NI) or *M. tuberculosis*-infected (HN878) mice. (C,D) Represented are Mean±SEM. Each dot represents an individual mouse, distributed in two independent experiments. The infection doses (in CFU±SEM) / animal ages (in weeks old at the time of infection) for the experiments used in thisfigure were 683±125 / 10; 1093±100 / 8; and 1057±74 / 9 and 10. Non- infected animals were 8 weeks old. The statistical analyses were performed using Mann–Whitney U test to identify statistical differences between groups. p-values inferior to 0.05 were deemed significant: * p-value < 0.05. Alv. M0, alveolar macrophages; DCs, dendritic cells; Rec. M0, recruited macrophages.

**
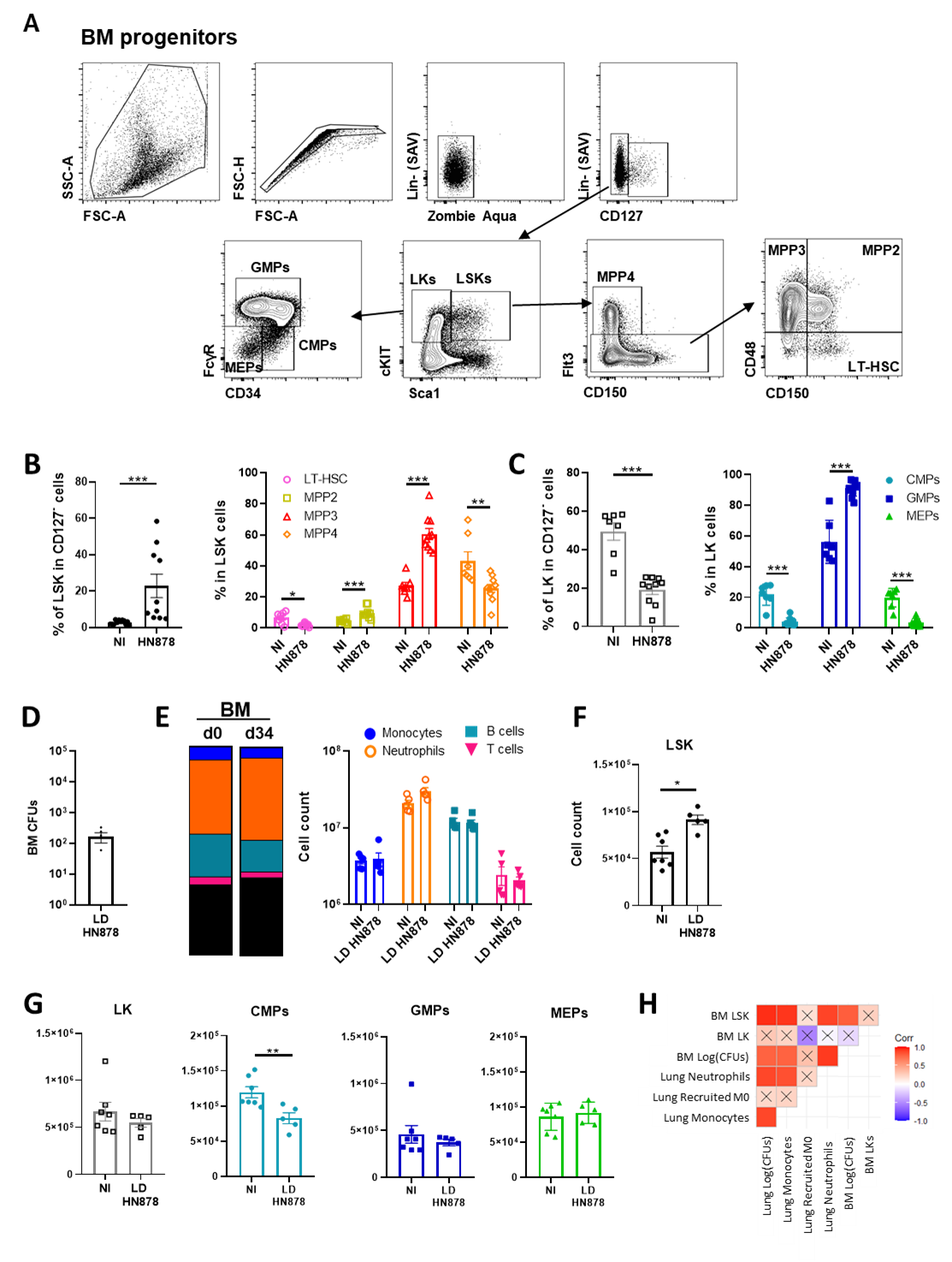
**

**Supplementary Figure 2.** (A) Gating strategy used to analyse bone marrow progenitor and precursor cell populations. (B-C) Frequencies of bone marrow precursor (B) and progenitor (C) cell populations in non-infected (NI) mice or mice infected with high doses of *M. tuberculosis* HN878. (D-G) C57BL/6 mice were infected via aerosol with low doses (LD) of *M. tuberculosis* HN878 isolate. On day 34 post-infection their bone marrow was analysed for bacterial burden (D), mature (E), progenitor (F) and precursor (G) cell populations. (H) Plot of Spearman’s correlation between Log10(CFUs), numbers of lung immune cell populations (monocytes, recruited macrophages and neutrophils), bone marrow CFUs, LSK and LK cell populations. Data used are from mice infected with low dose or high dose of *M. tuberculosis* HN878 for 34 or 27 days, respectively. Non-significant correlations are identified with a cross. (B-G) Represented are Mean±SEM. Each dot represents an individual mouse, distributed in two independent experiments. The infection doses (in CFU±SEM) / and animal ages (in weeks old at the time of infection) for the experiments used in this figure were: 1093±100 / 8 and 650±110 / 8 for high dose infections; 185±10 / 12 for low dose infection. Non-infected animals were 8 weeks old. The statistical analyses were performed using Mann–Whitney U test to identify statistical differences between groups. p-values inferior to 0.05 were deemed significant: * p-value < 0.05; ** p-value<0.01 and *** p-value < 0.001.

**
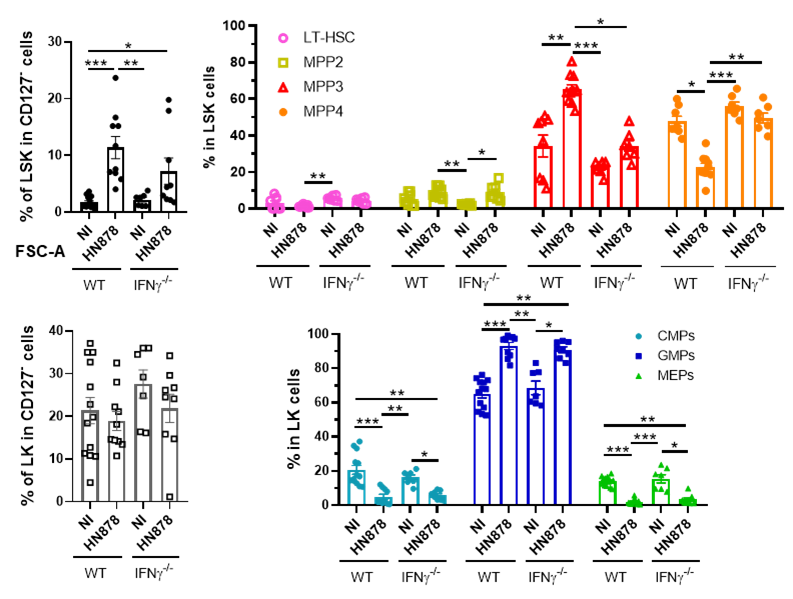
**

**Supplementary Figure 3.** Frequencies of bone marrow precursor and progenitor cell populations in C57BL/6 WT or IFNγ deficient non-infected (NI) mice or mice infected with high doses of *M. tuberculosis* HN878. Represented are Mean±SEM. Each dot represents an individual mouse, distributed in two independent experiments. The infection doses (in CFU±SEM) / and animal ages (in weeks old at the time of infection) for the experiments used in this figure were 1093±100 / 8 for WT and 11-12 for IFNγ^-/-^; and 1057±74 / 9-10 for WT and 9 for IFNγ^-/-^. Non- infected animals were 10-11 weeks old. The statistical analyses were performed using Kruskal–Wallis one-way analysis of variance to identify statistical differences between groups. p-values inferior to 0.05 were deemed significant: * p-value < 0.05, ** p-value < 0.01 and *** p-value < 0.001.

# Supplementary Table 1. List of antibodies used in this study.

| Flow cytometry – Mouse bone marrow, blood, and lung – mature immune cells | | |
| --- | --- | --- |
| Target | Clone | Company (Catalog #) |
| CD45 | 30-F11 | BioLegend (103112) |
| CD11c | N418 | BioLegend (117318) |
| CD11b | M1/70 | BioLegend (101245) |
| Ly-6G | 1A8 | BioLegend (127616) |
| Ly-6C | HK1.4 | BioLegend (128014) |
| CD3 | 145-2C11 | BioLegend (100308) |
| CD4 | GK1.5 | BioLegend (100453) |
| CD8 | 53-6.7 | BioLegend (100741) |
| CD19 | 6D5 | BioLegend (115530) |
| Lineage depletion – Mouse bone marrow precursors (Lin^+^) | | |
| Target | Clone | Company (Catalog #) |
| CD3 | 145-2C11 | BioLegend (100304) |
| CD4 | GK1.5 | BioLegend (100404) |
| CD8 | 53-6.7 | BioLegend (100704) |
| CD11c | N418 | BioLegend (117304) |
| CD11b | M1/70 | BioLegend (101204) |
| GR1 | RB6-8C5 | BioLegend (108404) |
| CD19 | 6D5 | BioLegend (115504) |
| B220 | RA3-6B2 | BioLegend (103204) |
| Nk1.1 | PK136 | BioLegend (108704) |
| TER-119 | TER-119 | BioLegend (116204) |
| Flow cytometry – Mouse bone marrow progenitors (Lin^-^) | | |
| Target | Clone | Company (Catalog #) |
| Ly-6A/E (Sca-1) | E13-161.7 | BD Pharmigen (553335) |
| CD16/CD32 (FcγR) | 2.4G2 | BD Pharmigen (560540) |
| CD48 | HM48-1 | BioLegend (103412) |
| CD117 (c-kit) | 2B8 | BioLegend (105825) |
| CD34 | RAM34 | Invitrogen (48-0341-82) |
| CD150 | TC15-12F12.2 | BioLegend (115941) |
| CD135 (Flt3) | A2F10 | BioLegend (135305) |
| CD127 (IL-7Rα) | A7R34 | BioLegend (135014) |
| Streptavidin (SAV) |  | BioLegend (405249) |

#

# Supplementary Table 2. List of oligonucleotides used in this study.

| Gene | Forward (5’-3’) | Reverse (5´-3’) |
| --- | --- | --- |
| *Csf1* | GTGTCAGAACACTGTAGCCAC | TCAAAGGCAATCTGGCATGAAG |
| *Csf3* | ATGGCTCAACTTTCTGCCCAG | CTGACAGTGACCAGGGGAAC |
| *Tnf* | GCCACCACGTCTTCTGTCT | TGAGGGTCTGGGCCATAGAAC |
| *Il1b* | ACCTTCCAGGATGAGGACATGA | AACGTCACACACCAGCAGGTTA |
| *Il6* | ACACATGTTCTCTGGGAAATCGT | AAGTGCATCATCGTTGTTCATACA |
| *Ifng* | CAACAGCAAGGCGAAAAAGG | GGACCACTCGGATGAGCTCA |
| *Ifnb* | GCACTGGGTGGAATGAGACT | AGTGGAGAGCAGTTGAGGACA |
| *Ubiquitin* | TGGCTATTAATTATTCGGTCTGCAT | GCAAGTGGCTAGAGTGCAGAGTAA |

#

**Supplementary Table 3. Pathological scoring analysis of HE-stained lung histological sections.**

| **Parameter** |  |
| --- | --- |
| **Lesion** | **Description** |
| 0 | Absence of lesions or alterations in the lung |
| 1 | Lung with onset of lesion:   - One or few very small infiltrates of inflammatory cells; - Composed of myeloid cells; - Perivascular location; - No other lesion signs present. |
| 2 | Lung with minor lesion:   - One or few small but well-formed infiltrates of inflammatory cells; - Composed mainly of myeloid cells such as macrophages and some lymphoid cells; - Perivascular location; - No other lesion signs present. |
| 3 | Lung with moderate lesion:   - One or few medium-sized lesions or small multifocal lesions; - Composed of cells such as macrophages/MN, neutrophils, and lymphocytes; - May present congestion. |
| 4 | Lung with extensive lesion:   - Several medium-sized lesions or a few very extensive ones; - Composed of various types of inflammatory cells; - Presence of some areas of necrosis or cellular debris and/or congestion. |
| 5 | Lung with very extensive lesion:   - Lung almost entirely covered in lesions; - Composed of various types of inflammatory cells; - Zones of necrosis in the center of the lesions and other damage elements such as congestion, edema and damaged epithelium. |
| **Necrosis/**  **Cell death** | **Description** |
| 0 | Absence of necrotic zones or cellular death. |
| 1 | Small/few localized zones with necrosis. |
| 2 | Lung with extensive necrosis. |
| **Exudate/ Pulmonary edema** | **Description** |
| 0 | Lung without edema. |
| 1 | Small areas with fluid/pulmonary edema. |
| 2 | Lung with vast areas of edema. |
| **Fibrosis/ Calcification** | **Description** |
| 0 | Absence of fibrosis and calcification. |
| 1 | Presence of fibrosis or calcification in an area of the lung. |
| 2 | Presence of fibrosis or calcification in more than one area of the lung. |
| **Final Score** | **Sum of the score for each parameter** |
